# Factors associated with the perception that eHealth facilitates access to healthcare

**DOI:** 10.1093/eurpub/ckac129.300

**Published:** 2022-10-25

**Authors:** E Kainiemi, L Virtanen, P Saukkonen, T Vehko, AM Aalto, AM Kaihlanen

**Affiliations:** Finnish Institute for Health and Welfare, Helsinki, Finland

## Abstract

**Background:**

The provision of eHealth has increased in the COVID-19 era with the aim of improving access to care without the risk of infection. Perceiving eHealth beneficial may affect the use. This study examined which patient-related factors are associated with the perception that eHealth facilitates access to care.

**Methods:**

A nationwide survey was sent to 61,600 Finnish residents during the COVID-19 pandemic (September 2020-February 2021). Binary logistic regression analysis was used to examine whether the service needs (self-rated health, met service needs, challenges accessing traditional care) and eHealth experience (e-visits, need for guidance, data security concerns) were associated with perceiving that eHealth facilitates access to care. The model was adjusted for age, gender and education.

**Results:**

The study included 21,409 respondents who had used healthcare services in the past 12 months (55.0% female, mean age 52.88, SE .18). The majority (63.8%) agreed with the benefit that eHealth facilitates access to care. Patients in good health (OR 1.24, 95% CI 1.13-1.37), whose service needs were met (OR 1.34, 95% CI 1.19-1.52) and who had no challenges in accessing traditional care (OR 1.18, 95% CI 1.02-1.36) had greater odds of perceiving the benefit compared to their counterparts. Patients with experience of e-visits (OR 2.60, 95% CI 2.28-2.96) and without need for guidance (OR 1.71, 95% CI 1.52-1.91) had greater odds of perceiving the benefit compared to their counterparts.

**Conclusions:**

Patients in good health, with met service needs and with easy access to traditional care appear to perceive eHealth more beneficial than their counterparts, which might exacerbate the already existing inequalities in healthcare access and health outcomes. Promotion of eHealth skills might increase equitable opportunities to benefit from eHealth for those patients whose service needs could be met without traditional face-to-face encounter.

**Key messages:**

• Patients in good health, with met service needs and without difficulties accessing traditional healthcare appear to find eHealth more beneficial than their underprivileged counterparts.

• Promotion of eHealth skills might increase equitable opportunities to benefit from eHealth for those patients whose service needs could be met without traditional face-to-face encounter.

